# Imaging Inflammation in Atherosclerosis with CXCR4-Directed [^68^Ga]PentixaFor PET/MRI—Compared with [^18^F]FDG PET/MRI

**DOI:** 10.3390/life12071039

**Published:** 2022-07-12

**Authors:** Xia Lu, Raffaella Calabretta, Wolfgang Wadsak, Alexander R. Haug, Marius Mayerhöfer, Markus Raderer, Xiaoli Zhang, Jingle Li, Marcus Hacker, Xiang Li

**Affiliations:** 1 Division of Nuclear Medicine, Department of Biomedical Imaging and Image-Guided Therapy, Medical University of Vienna, 1090 Vienna, Austria; lxgf2222@163.com (X.L.); raffaella.calabretta@meduniwien.ac.at (R.C.); wolfgang.wadsak@meduniwien.ac.at (W.W.); alexander.haug@meduniwien.ac.at (A.R.H.); ljle2012@163.com (J.L.); marcus.hacker@meduniwien.ac.at (M.H.); 2 Department of Nuclear Medicine, Northern Jiangsu People’s Hospital, Yangzhou 225001, China; 3 Department of Nuclear Medicine, Laboratory for Molecular Imaging, Beijing Anzhen Hospital, Capital Medical University, Beijing 100029, China; xiaolizhang68@126.com; 4 Center for Biomarker Research in Medicine, CBmed, 8036 Graz, Austria; 5 Department of Radiology, Memorial Sloan Kettering Cancer Center, 1275 York Avenue, New York, NY 10065, USA; marius.mayerhoefer@meduniwien.ac.at; 6 Division of General and Pediatric, Radiology, Department of Biomedical Imaging and Image-Guided Therapy, Medical University of Vienna, 1090 Vienna, Austria; 7 Department of Medicine I, Division of Oncology, Medical University of Vienna, 1090 Vienna, Austria; markus.raderer@meduniwien.ac.at

**Keywords:** atherosclerosis, inflammation, CXCR4, [^68^Ga]PentixaFor, PET/MRI

## Abstract

(1) This study compared [^68^Ga]PentixaFor uptake in active arterial segments with corresponding [^18^F]FDG arterial uptake as well as the relationship with cardiac [^68^Ga]PentixaFor uptake. (2) Method: Tracer uptake on atherosclerotic lesions in the large arteries was measured and target-to-background ratios (TBR) were calculated to adjust background signals with two investigators blinded to the other PET scan. On a patient-based and lesion-to-lesion analysis, TBR values of two tracers were compared and the relationship with cardiac inflammation was further explored. Furthermore, two cardiovascular risk-related groups were divided to explore the value of risk stratification of the two tracers in atherosclerosis. (3) Results: [^68^Ga]PentixaFor PET/MRI identified more lesions (88% vs. 48%; *p* < 0.001) and showed higher uptake than [^18^F]FDG PET/MRI (TBR, 1.90 ± 0.36 vs. 1.63 ± 0.29; *p* < 0.001). In the patient-based analysis, the TBR of [^68^Ga]PentixaFor uptake was also significantly higher than [^18^F]FDG uptake (1.85 ± 0.20 vs. 1.42 ± 0.19; *p* < 0.001). The TBR of active lesions for [^68^Ga]PentixaFor was significantly increased in the high-risk group (*n* = 9), as compared to the low-risk group (*n* = 10) (2.02 ± 0.15 vs. 1.86 ± 0.10, *p* = 0.015), but not for [^18^F]FDG (1.85 ± 0.10 vs. 1.80 ± 0.07, *p* = 0.149). (4) Conclusion: [^68^Ga]PentixaFor PET/MRI identified many more lesions than [^18^F]FDG PET/MRI. Patients with high-risk cardiovascular factors illustrated an increased uptake of [^68^Ga]PentixaFor. There was a correlation between the elevated uptake of [^68^Ga]PentixaFor in the active arterial segments and heart.

## 1. Introduction

Atherosclerosis, a chronic and long-lasting arterial disease, is the leading cause of cardiovascular disease, including heart attack, organic ischemia, and stroke [1]. Atherosclerotic lesions can have decades of asymptomatic progression until atheroma plaques develop with clinical manifestation. The arterial lumen becomes stenotic gradually owning to the enlargement of plaque [2]. The subsequent alteration of blood flow and plaque rupture received the worse clinical outcomes [3], including thrombus formation, lumen occlusion, and ischemic events [4]. Chronic arterial inflammation is the primary underlying pathology of vulnerable atherosclerotic plaque (AS), which is prone to erosion and rupture and is responsible for life-threatening clinical endpoints [5,6]. A prominent feature of inflammation is the infiltration of immune cells such as T cells and macrophages in atherosclerosis [7].

Positron emission tomography/computed tomography (PET/CT) imaging is currently used for the visualization of biological information of vulnerable plaque in clinical and pre-clinical cardiovascular research. The [^18^F]FDG PET/CT has become a useful fusion technique for assessing large arterial inflammation noninvasively, and was proposed as a potential and feasible technique to assess plaque inflammation directly and to stratify patients by risk [8,9,10]. However, [^18^F]FDG merely reflects glucose metabolism information and may lack specificity to discriminate infection, inflammation, and neoplastic disease [11]. The CT component of vascular PET/CT imaging, on the other hand, unlike PET applications in whole-body imaging, often lacks sufficient clinically valuable information beyond attenuation correction, but a CT scan does add to the radiation dosage. Therefore, more specific tracers targeting the specific cells and molecules involved in atherosclerosis and more sensitive imaging techniques need to be developed and investigated.

C-X-C motif chemokine receptor 4 (CXCR4) is a transmembrane chemokine receptor involved in mediating pro-inflammatory leukocytes and is particularly abundantly expressed on monocytes, differentiated macrophages, and T cells in atherosclerosis [12]. CXCR4-directed PET imaging using [^68^Ga]PentixaFor has recently been demonstrated to accurately identify the inflammation of atherosclerotic plaque by targeting human CXCR4 expression in vivo [13]. Our previous pilot study evaluated the arterial [^68^Ga]PentixaFor uptake in correlation with arterial stenosis level in a PET/CT imaging study [14]. 

The hybrid of PET and magnetic resonance imaging (MRI) is the optimal method available to date. Combined PET/MRI scans acquire simultaneous high-quality data about anatomical information, as well as early biological changes, to ensure the sensitive detection of disease and lower radiation exposure for patients. PET could be used to differentiate the high uptake of inflammation from vulnerable plaques on the aorta from stable plaques with a low uptake of tracer at the initial work-up, providing a precise prognosis for patients with a high rate of major adverse cardiovascular events (MACE), and treatment decision making. T1-weighted imaging (T1WI), T2-weighted images (T2WI)/proton density-weighted images (PDWI), and time of flight (TOF) sequences are all useful in assessing atherosclerosis, as well as the whole-body evaluation of cardiovascular disease [15,16]. PET attenuation correction methods based on MR imaging are more sophisticated than for CT attenuation correction methods, but they are possible and entail tissue segmentation algorithms [17].

In this study, we aimed to compare arterial [^68^Ga]PentixaFor and [^18^F]FDG uptake using PET/MRI, and the correlations between regional radioactivity and cardiovascular risk factors, as well as myocardial signaling.

## 2. Materials and Methods

### 2.1. Study Population

Nineteen oncological patients (lymphoma) with visible focal uptake on the arterial wall were assessed in this retrospective analysis with signed written informed consent. All patients underwent [^68^Ga]PentixaFor PET/MR and [^18^F]FDG PET/MR imaging within one week for staging or restaging of the malignancy. None of these patients received steroids or had a recent history of inflammation or vasculitis.

According to cardiovascular risk factors, all patients were divided into the high-risk group (patients with DM (*n* = 2), dyslipidemia (*n* = 4), hypertension (*n* = 4), high CRP level (*n* = 2), and smoking (*n* = 5)) and the low-risk group (patients without any cardiovascular risk factor).

### 2.2. Imaging Procedures

[^68^Ga]Pentixafor was produced in an automated procedure using a GRP module. The tracer was purified with a C18 solid-phase extraction cartridge and eluted with ethanol/water, subsequently formulated in PBS buffer. All patients underwent [^68^Ga]PentixaFor PET/MRI and [^18^F]FDG PET/MRI on a dedicated PET/MRI scanner (Biograph mMRI, Siemens Healthcare GmbH, Erlangen, Germany). As for the [^18^F]FDG PET imaging, patients fasted for at least 6 h to ensure a serum glucose level below 130 mg/dL. An amount of 10 mg of furosemide was administered intravenously concurrently with the injection of FDG (4.18 ± 0.88 MBq/kg bodyweight). After an uptake period of 60 to 90 min, transmission data were acquired, extending from the base of the skull to the proximal thighs. PET images were acquired for at least five bed positions with 5 min per bed position. The MR imaging was performed with an integrated radiofrequency coil and a multi-station protocol. The slice thickness was 2 mm. 

The PET emission data were acquired in a three-dimensional mode with a 200 × 200 matrix with a 2 min emission time per bed position. The implemented standard four-compartment model attenuation map was derived from a Dixon-based VIBE (volumetric interpolated breath-hold examination) sequence for attenuation correction. A 3D ordinary Poisson ordered subsets expectation maximization (OP-OSEM) algorithm with PSF correction and 3 iterations and 21 subsets was used for reconstruction. The image matrix size was 172 × 172 (pixel size 4.2 mm). The reconstructed images were subsequently smoothed with a 3-mm fullwidth at a half-maximum (FWHM) Gaussian filter.

As for the [^68^Ga]PentixaFor PET/MR imaging, patients were injected with 165 ± 29 MBq (range: 78 to 229 MBq) of [^68^Ga]PentixaFor, the acquisition was started after 40 to 60 min with the same parameters as mentioned above.

### 2.3. Imaging Analysis

PET/MR images were reconstructed and analyzed as previously described [14]. [^68^Ga]PentixaFor and [^18^F]FDG uptake were visually and semi-quantitatively assessed with commercially available software (Hermes Hybrid 3D, Hermes Medical Solutions, Stockholm, Sweden) in trans-axial PET, MRI, and PET/MRI slices, respectively. All axial PET image slices were inspected visually, along with eight arterial segments, including the left and right carotid arteries, aortic arch, ascending and descending aorta, abdominal aorta, and left and right iliac arteries. Signal intensities in target regions were quantified using a maximum SUV (SUV_max_), obtained by manually placing an individual circular volume of interest (VOI) around atherosclerotic lesions. For the semi-quantitative analysis, the whole heart VOI was derived from every axial PET image slice of ROI with maximum cardiac uptake. As a reference, the background was defined as the average blood-pool uptake as determined by the mean SUV of three different ROIs (diameter of 1 cm) within the lumen of the vena cava. The TBR of each arterial segment was calculated as the ratio of SUV_max_ measured in the arterial wall corrected with SUV_mean_ in the venous blood pool [9]. Arterial segments with TBR threshold of >1.6 were considered as active in both scans, as described previously [18]. The TBR of each patient was defined as the average TBR of all active lesions in both scans. 

### 2.4. Statistical Analysis

The Statistical Package for Social Sciences (SPSS version 11.0; SPSS Inc. (IBM), Armonk, NY, USA) was used for statistical analyses. Continuous variables with a normal distribution were recorded as mean ± standard deviation. Paired t-tests were used to compare uptake (ratios) of corresponding lesions and patients between the two tracers. Pearson correlation coefficients were used to assess the association of the arterial uptake activity of [^68^Ga]PentixaFor and [^18^F]FDG between the atherosclerotic plaques and the myocardium. *p* values < 0.05 were considered statistically significant.

## 3. Results

### 3.1. Comparison of [^68^Ga]PentixaFor and [^18^F]FDG Uptake: Lesion-Based Analysis

**Figure 1:**
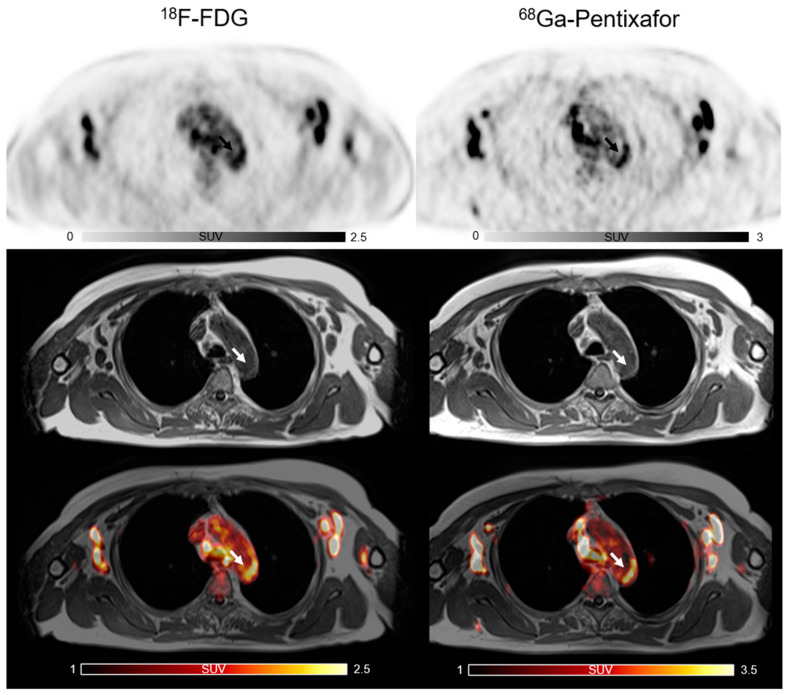
The co-localized focal vascular uptake of both [^18^F]FDG and [^68^Ga]PentixaFor (white arrow) in active atherosclerotic lesions.

**Figure 2:**
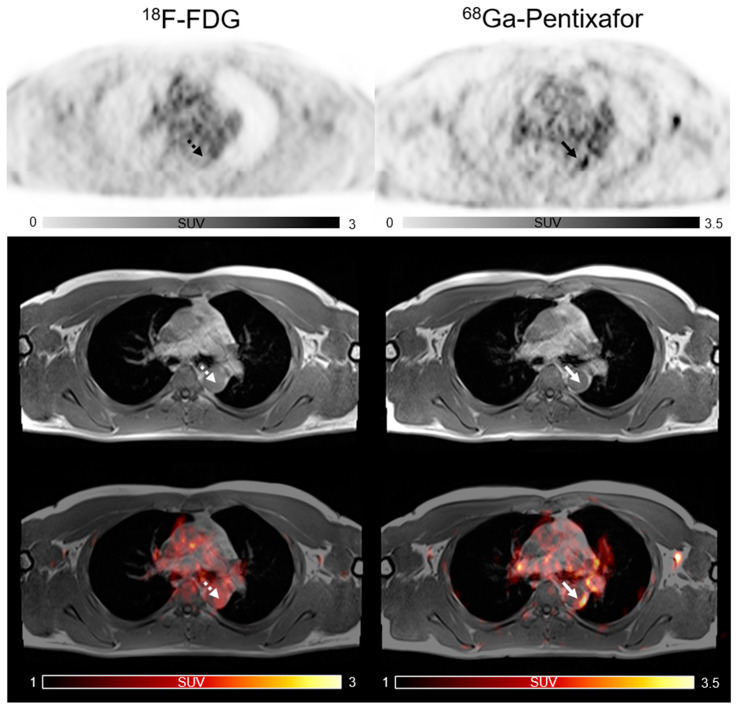
Focal vascular uptake of [^68^Ga]PentixaFor (white arrow) but without corresponding focal [^18^F]FDG uptake in active lesions (white dotted arrow).

A total of 887 sites of active lesions (TBR > 1.6) were identified. Three patterns of uptake in arterial walls were found in PET/MR images of the [^68^Ga]PentixaFor and [^18^F]FDG PET/MR images. The lesions with co-localized uptake of two tracers are shown in Figure 1 and the individually positive detection of [^68^Ga]PentixaFor or [^18^F]FDG uptake results are depicted in Figure 2. In 781 active lesions of [^68^Ga]PentixaFor, a co-localized active [^18^F]FDG uptake was observed in 48% (374 sites), while 52% (407 sites) were discordant. Of 480 active lesions of [^18^F]FDG, as many as 78% (374 sites) had an increased uptake of [^68^Ga]PentixaFor, while only 22% (106 sites) were discordant. 

**Figure 3:**
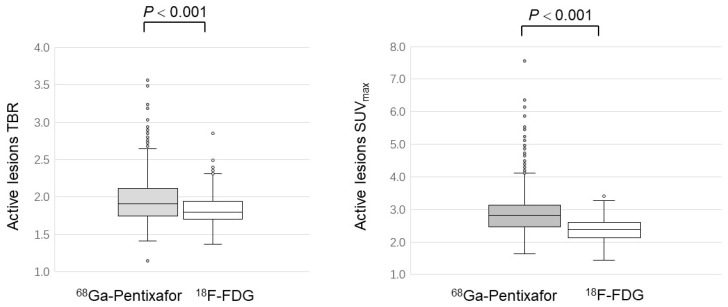
Comparison of [^68^Ga]PentixaFor and [^18^F]FDG uptake in active atherosclerotic lesions.

As for the SUV_max_ and TBR of all active lesions, [^68^Ga]PentixaFor displayed a significantly higher value than [^18^F]FDG (SUV_max_: 2.88 ± 0.64 versus 2.36 ± 0.36; *p* < 0.001; TBR:1.90 ± 0.36 versus 1.63 ± 0.29; *p* < 0.001) (Figure 3). 

### 3.2. Comparison of [^68^Ga]PentixaFor and [^18^F]FDG Uptake: Patient-Based Analysis 

Baseline demographics and clinical characteristics of the study population are summarized in Table 1. In the patient-based analysis, the mean SUV_max_ (2.84 ± 0.82 versus 1.85 ± 0.30; *p* < 0.001) and mean TBR (1.85 ± 0.20 versus 1.42 ± 0.19; *p* < 0.001) of [^68^Ga]PentixaFor were significantly higher than those of [^18^F]FDG. 

**Figure 4:**
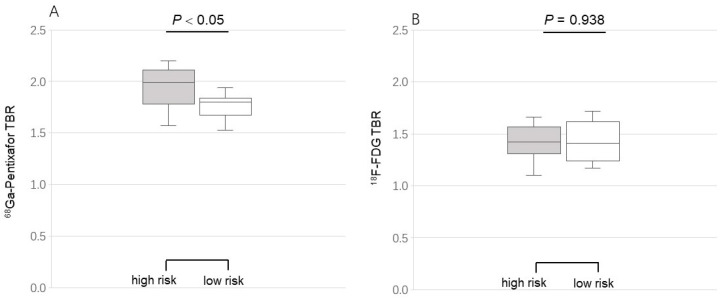
Comparison of [^68^Ga]PentixaFor (**A**) and [^18^F]FDG (**B**) uptake grouping by the cardiovascular risk factors. Target-to-background ratios (TBR) of [^68^Ga]PentixaFor (not [^18^F]FDG) uptake in the high-risk group were significantly higher than those of TBR in the low-risk group.

Two risk-related groups were set to assess the difference in activity of the two tracers. The mean TBR of active lesions for [^68^Ga]PentixaFor was significantly increased in the high-risk group (*n* = 9), as compared to the low-risk group (*n* = 10) (2.02 ± 0.15 vs. 1.86 ± 0.10, *p* = 0.015), but not for [^18^F]FDG (1.85 ± 0.10 vs. 1.80 ± 0.07, *p* = 0.149) (Figure 4). Both tracers’ TBR showed no correlation with the clinical risk factors.

### 3.3. Pearson Correlation between Tracer Uptake of Plaques and Myocardial CXCR4 Expression

Regarding the systemic inflammatory response, the TBR of [^68^Ga]PentixaFor in atherosclerotic plaques were significantly positively correlated with myocardial uptake (r = 0.53, *p* = 0.019), but not in [^18^F]FDG (r = 0.073, *p* = 0.77).

## 4. Discussion

In this study, we compared the performance of [^68^Ga]PentixaFor PET/MRI for imaging atherosclerosis with [^18^F]FDG PET/MRI. We found that [^68^Ga]PentixaFor identified more active lesions of atherosclerosis than [^18^F]FDG, which was consistent with our previous study with PET/CT [9] and illustrated that [^68^Ga]PentixaFor might be more sensitive than [^18^F]FDG in assessing atherosclerotic plaques. In addition, the high uptake of [^68^Ga]PentixaFor and [^18^F]FDG in atherosclerotic plaques was weakly correlated in 887 atherosclerotic lesions. In addition, the average TBR of [^68^Ga]PentixaFor in the active segments was significantly higher than that for [^18^F]FDG. Meanwhile, in patient-based analysis, [^68^Ga]PentixaFor uptake on atherosclerosis (corrected by blood pool) was significantly increased in the high-risk patients, as compared to the low-risk patients, indicating a potential value of elevated CXCR4 expression in the risk stratification of patients with atherosclerosis.

Our previous comparative study with larger patient cohorts mainly demonstrated the correlation between radio-uptake and calcification extent. In this present study, we sub-grouped the patients into high cardiovascular risk and low risk, demonstrating the potential of [^68^Ga]PentixaFor PET/MRI in patients’ risk stratification. In addition, we firstly analyzed the relationship between [^68^Ga]PentixaFor uptake in active arterial segments and cardiac uptake. This finding indicated symmetric CXCR4 immune signaling along with the cardiovascular system.

Atherosclerosis is considered a systemic and complex inflammatory disease involved in multiple factors and cells, based on recent studies. Atherosclerosis has been proven to be the main reason for the development of coronary artery disease (CAD), as well as carotid disease, causing the most mortality and morbidity in the world [19]. Common risk factors of atherosclerosis in the clinic mainly include old age, being male, smoking, obesity, high systolic blood pressure (hypertension), high-density lipoprotein cholesterol and total cholesterol content (dyslipidemia), diabetes mellitus, and left ventricle hypertrophy. However, the true etiology and mechanisms of atherosclerotic plaque pathogenesis are still poorly understood and research into a combination of additional, and as yet unknown, factors could help us better understand this disease. Based on the research of radiolabeled tracers, the PET technique could provide highly sensitive information to quantify several important pathological events in vulnerable atherosclerosis, including plaque composition, inflammation, calcification, and other characteristics. Owing to an in-depth understanding of the atherosclerotic disease pathway nowadays, many innovative radiotracers have been designed and synthesized to target detecting the pathophysiological characteristics of high-risk vulnerable plaques, allowing for earlier intervention opportunities [20]. Unfortunately, the PET resolution is low, and equipment with better spatial resolution, such as MRI, must be infused with PET images to translate the novel results of vulnerable plaques from bench to bedside. Hybrid PET/MR imaging is particularly promising in atherosclerosis molecular imaging because it allows soft-tissue characterization and molecular function to be traced simultaneously with a low radiation dose and comparable agreement with PET/CT, as reported in several recent studies [21,22,23].

Osamu et al. revealed that CXCR4 expression was not only limited to macrophages in atherosclerosis, but was also induced on the endothelium at the margins of plaques where endothelial permeability and proliferation are prominent. Furthermore, their data suggested that macrophages in plaques may be inaccessible for tracers to target due to inadequate access of the tracer to the deep regions of the vulnerable plaque, whereas the endothelium is quite easily accessible and, thus, obviously shows a signal [24]. Thereby, [^68^Ga]PentixaFor PET/MRI could also detect active lesions that were inconspicuous in [^18^F]FDG PET/MRI and the source of the PET signal likely originates from additional cell types beyond mere inflammation [18]. The presence of [^68^Ga]PentixaFor positive uptake might indicate an atherosclerosis acceleration. In addition, compared to PET/CT, a comprehensive characterization of atherosclerosis might benefit from hybrid PET/MRI to delineate the arterial wall and the activity of atherosclerotic lesions based on the higher soft-tissue contrast and spectroscopic information of MRI [25,26]. Fabiano et al. found that the overall sensitivity and specificity rates of 1.5 T MRI for the lipid core were 92% and 74%, respectively, for the fibrous tissue were 82% and 94%, for the fibrous/loose connective tissue were 72% and 87%, and for calcification were 98% and 99%, respectively. Therefore, an MRI instead of a CT combined with PET proved to be a more reliable tool to characterize vulnerable plaque components and to help screen high-risk patients with worse clinical outcomes [27]. The possibility of applying PET/MRI in routine clinical practice could change the non-invasive approach to artery diagnostic imaging, thus allowing the early identification of patients with vulnerable plaques to achieve good clinical outcomes.

Recent studies described a potential and important value of CXCR4 on atherosclerosis. CXCR4 expression was obviously elevated by the macrophage-colony-stimulating factor to progress atherosclerosis, indicating a pro-atherogenic role of CXCR4 in atherosclerosis and a potentially promising therapy of inhibiting CXCR4 expression in vulnerable atherosclerotic plaques with high inflammatory activity. Additionally, the accumulation of [^68^Ga]PentixaFor, especially in plaques with inflammation, was verified by histological staining in human carotid plaques and in a rabbit model, respectively [13]. Iiza et al. validated that CXCR4 could contribute to the later stages of plaque progression by perturbing neutrophil function [28]. These findings indicate the potential value of [^68^Ga]PentixaFor PET/MRI for targeting CXCR4 expression in atherosclerosis to monitor anti-inflammation therapy [29]. We also found that patients with cardiovascular risk factors illustrated an increased uptake of [^68^Ga]PentixaFor in PET/MRI. Although extensive investigations have explored the role of CXCR4 expression in atherosclerosis, the basis of its metabolism is still incompletely understood. Therefore, CXCR4-directed imaging of atherosclerotic inflammation with [^68^Ga]PentixaFor PET/MRI in high-risk patients would warrant a promising study in monitoring progression and anti-inflammation therapy in atherosclerotic plaques.

CXCR4 expressed on macrophages induced endothelial activation by the increased expression of adhesion molecules and the release of pro-inflammatory cytokines, which recruits infiltrating inflammatory cells. The CXCR4/CXCL12 axis also seems to play a key role in cardiogenesis and cardiomyocyte calcium homeostasis regulation and was shown to be involved in the neovascularization of an injured heart [30], which induced the concordant high uptake of [^68^Ga]PentixaFor in atherosclerotic plaques and heart in our study. In cardiovascular diseases, the relevance of systemic causes of disease development, progression, and treatment response is widely recognized. Chronic systemic inflammation on the arterial wall [31] can induce heart dysfunction, and this cardiovascular CXCR4 axis might play a vital role in understanding diseases. However, the mechanism of CXCR4 for systematic cardiovascular and inflammatory activation in patients with atherosclerosis is yet to be elucidated.

Furthermore, considering that [^68^Ga]PentixaFor PET/MRI was not much more expensive than [^18^F]FDG PET/MRI in clinical practice, its cost-effectiveness for the diagnosis of vulnerable plaque is favorable for the management of therapy strategy.

## 5. Limitations

This retrospective study has several limitations. Firstly, this study concerns an oncologic cohort, which might limit the diagnostic accuracy of the results, as tracer circulation time after injection would affect the semi-quantification of plaque uptake. Furthermore, we could not remove the biological processes of CXCR4, underlying malignancy, and the hormone response of immune-modulatory drugs, and anticancer therapies may have also influenced the accuracy of results. The lack of dedicated MRI sequences performed for the aortic wall imaging was another limitation in this study. The accurate allocation and characterization of arterial segments and components were challenging. We also could not ignore the significant limitation of partial volume effects in small-sized atherosclerotic lesions. In addition, the high positron energy and high positron range of gallium-68 lead to noisier images and a poorer spatial resolution compared to fluorine-18, which is amplified by the lower injected activity. This limitation is partly compensated for by the predominantly high specificity of most [^68^Ga]-labeled radiopharmaceuticals in comparison to the non-specific tissue uptake of [^18^F]-labeled FDG [32]. Another limitation was that we defined the TBR cutoff as 1.6 for [^18^F]FDG active segments, which was proposed by an expert committee [33], but the same TBR cutoff was used for [^68^Ga]PentixaFor, which might result in potential false-positive findings. In addition, this study lacks an evidential standard for a true-positive uptake for the vascular wall, and the two tracers are compared by visual analysis. This also may result in a possible false-positive uptake of [^68^Ga]PentixaFor. Lastly, unfortunately, the vessel-dedicated contrast MRI was not performed in a hybrid PET/MRI system as the standard approach for the vessel wall.

## 6. Conclusions

In this study, we found that [^68^Ga]PentixaFor PET/MRI identified more active arterial segments as compared to [^18^F]FDG. Patients with high-risk cardiovascular factors demonstrated higher [^68^Ga]PentixaFor activity in PET/MR imaging. There was a significant correlation between active segmental uptake and corresponding cardiac uptake. Further studies to elucidate the underlying systematic biological mechanism and its translation to clinic are highly warranted.

## Figures and Tables

**Table 1:** Baseline demographics and clinical characteristics of the study population.

**Patient Characteristics (*n* = 19)**	
Age (y)	68 ± 10
Gender	11 male/8 female
BMI (kg/m^2^)	27.1
**Cardiovascular Risk Factors** (*n*, %)	
Hypertension	4 (21%)
Dyslipidemia	4 (21%)
Diabetes	2 (11%)
Smoking	5 (26%)
CRP (≥3 mg/L)	2 (11%)
